# Effects of Xuezhitong in Patients with Hypertriglyceridemia: a Multicentre, Randomized, Double-Blind, Double Simulation, Positive Drug and Placebo Parallel Control Study

**DOI:** 10.1007/s10557-020-06965-3

**Published:** 2020-03-23

**Authors:** Wenhao Jia, Yan Li, Jie Wan, Xiaoyun Cui, Jinjin Lu, Jing Liu, Dong Li, Lei Li, Ting Zou, Junpin Ding, Qian Lin

**Affiliations:** 1 Beijing University of Chinese Medicine, Beijing, China; 2 Dongfang Hospital, Beijing University of Chinese Medicine, Beijing, China; 3 Technology Center for Drug Research and Evaluation, Chinese Association of Traditional Chinese Medicine, Beijing, China; 4 Beijing Compete Pharmaceutical Technology Development Co. LTD, Beijing, China; 5 Harbin kansaisi Pharmaceutical Technology Development co. LTD, Harbin, China; 6 Dongzhimen Hospital, Beijing University of Chinese Medicine, Beijing, China

**Keywords:** Xuezhitong, Hypertriglyceridemia, Efficacy, Safety, Clinical trial

## Abstract

**Backgroud:**

Xuezhitong (XZT) is an extract of Allium macrostemon Bunge that has lipid-lowering properties.

**Objective:**

To evaluate the effects of XZT on lipids in subjects with hypertriglyceridemia (HTG) without severe dyslipidaemia.

**Methods:**

A total of 358 subjects with HTG were enrolled and randomly assigned to receive XZT (2700 mg daily), xuezhikang (XZK) (1200 mg daily) or placebo. The primary endpoint was the reduction or percent reduction in the TG level over 12 weeks of treatment.

**Results:**

At the 12-week follow-up, a reduction in the TG level from baseline was observed in both groups, but the XZT and XZK groups demonstrated a significantly greater reduction than the placebo group (30.77%, 24.02% vs 11.59%, *P* < 0.0167); 70.54% of subjects in the XZT group and 62.30% of subjects in the XZK group demonstrated reductions in TG levels of at least 20%, compared with 41.67% of the subjects in the placebo group (*P* < 0.0167). Treatment with XZT capsules also demonstrated superior performance compared with the placebo with respect to the control of lipids (17.97% vs 5.00%), total cholesterol (TC) (14.18% vs 3.89%), low-density lipoprotein cholesterol (LDL-C) (17.98% vs 2.95%), and high-density lipoprotein cholesterol (HDL-C) (21.47% vs 2.16%). Daily use of XZT for 12 weeks resulted in statistically significant (65.22% vs 38.30%, 25.00%; *P* < 0.0167) and clinically meaningful increases in HDL-C levels by ≥4 mg/dl compared with XZK and placebo. XZT was safe and well tolerated; the safety and tolerability profiles were similar across treatment groups. No subject experienced myopathy or markedly elevated liver transaminases or creatine kinase.

**Conclusions:**

XZT significantly reduced TG levels and was well tolerated. Longer-term studies in more diverse patient populations are needed to corroborate these findings.

**Clinical Trial Registration:**

www.chictr.org.cn Identifier: ChiCTR1900025854.

## Introduction

Dyslipidaemia is a common metabolic disease that is closely related to the occurrence and development of atherosclerotic cardiovascular disease (ASCVD) [1]. Elevated low-density lipoprotein cholesterol (LDL-C) levels are considered to be independent risk factors for cardiovascular events. Statins can effectively reduce LDL-C and reduce the incidence of cardiovascular events, and they have become the cornerstone of ASCVD prevention and treatment. However, although the comprehensive control of traditional cardiovascular risk factors has been effective, after standard treatments guided by current clinical evidence, including the treatment of traditional risk factors such as smoking, unhealthy lifestyles, hypercholesterolaemia, hypertension, hyperglycaemia and obesity, patients are still at risk of major vascular and microvascular events; that is, the residual cardiovascular risk remains [2]. Hypertriglyceridaemia (HTG), the most common type of dyslipidaemia in China, is worthy of attention for its correlation with residual cardiovascular risk [3]. Studies have shown that patients with high TG levels (2.26–5.64 mmol/L) have higher residual cardiovascular risk, worse cardiovascular outcomes and higher overall medical costs after treatment with statins [4, 5], for every 1 mmol/L increase in TG, the risk of ASCVD increased by 13% [6]. Thus, Further reductions in ASCVD risk can potentially  be achieved with drugs that lower triglyceride levels [7] .

Xuezhitong (XZT) is a single-prescription traditional Chinese medicine that is enriched, purified and refined from the natural edible and medicinal plant Allium macrostemon Bunge, which is also known as Xie Bai. Xie Bai is an ancient Chinese medicinal plant that is widely used to treat CVDs in China. Modern pharmacological studies have shown that Allium macrostemon contains a variety of active ingredients, such as methyl allyl trisulfide, saponins, and adenosines [8]. Preliminary studies have proven that XZT can significantly reduce blood lipids, inhibit platelet aggregation, confer anti-thrombosis, and prevent and treat atherosclerosis [9]. Xuezhikang (XZK) is another natural lipid-lowering drug extracted from fermented red yeast rice, which contains lovastatin and statin homologue and a variety of essential amino acids, unsaturated fatty acid, sterol, and small amounts of flavonoids [10]. Studies have shown that the effect of XZK in reducing TC and LDL-C levels is equivalent to that of statins under conventional doses, and that it is better than statins in increasing HDL-C levels. More importantly, the treatment is more tolerable and safer than statins [11]. Xu et al. found that XZK can not only affect the level of blood lipids, but also significantly reduce atherosclerotic small LDL subfractions, oxidative stress and inflammatory markers [12].

The objectives of this study were to assess the effects of XZT (vs XZK and placebo) on TG and other blood lipids and to determine its tolerability and safety profiles in patients with HTG.

## Methods

### Study Design

This multicentre, randomized, double-blind, double simulation, positive drug and placebo parallel control trial was conducted at 17 sites in China between April 5, 2017, (first patient enrolled) and April 30, 2019 (final patient followed up). All the patients were out-patients, recruited from 17 sites by advertisement. The study was registered at www.chictr.org.cn (identifier ChiCTR1900025854). The flow chart of this study is shown in Table 1.

**Table 1:**
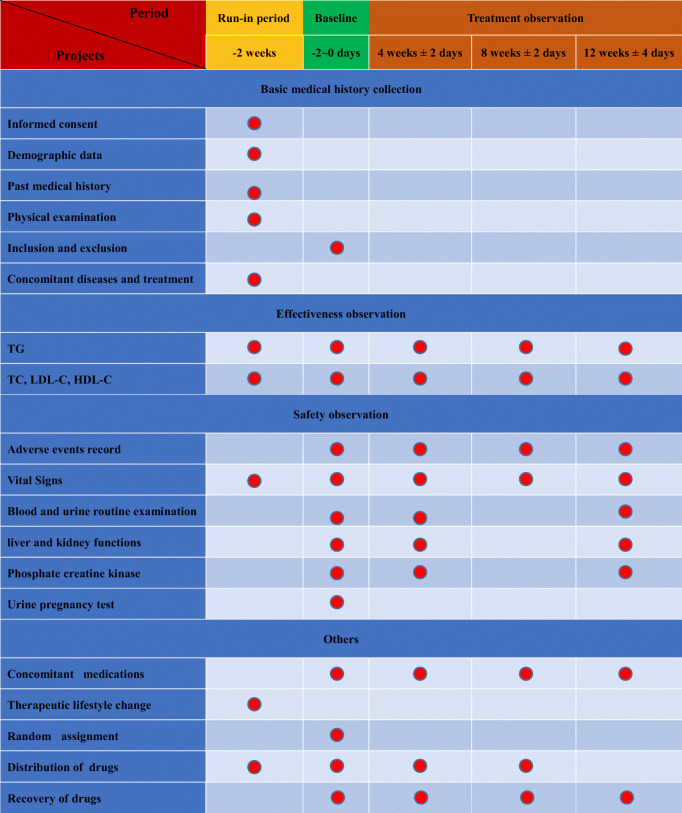
Trial flow chart

**“****”** means the project should be done in that period

### Subjects

Eligible subjects were aged 18 to 80 years with triglycerides (TG) > 2.3 mmol/L (but <6.5 mmol/L), LDL-C < 4.9 mmol/L and total cholesterol (TC) < 7.2 mmol/L after undergoing a 2-week therapeutic lifestyle change period. Other requirements were they followed a low-fat–modified diet. Patients were excluded if secondary hyperlipidaemia was caused by systemic diseases (such as nephrotic syndrome, hypothyroidism, liver disease or renal failure) or drugs (adrenal corticosteroids and some contraceptives, etc.) and if they had homozygous hypercholesterolemia. We excluded patients who had acute coronary syndrome, acute cerebrovascular diseases, diabetes mellitus can significantly impact TG and a systolic blood pressure ≥ 180 mmHg or a diastolic blood pressure ≥ 110 mmHg within 12 weeks. Other exclusion criteria were as follows: (1) peripheral atherosclerotic diseases and stenosis of arteries of more than 70%; (2) severe primary cardiac, cerebral, pulmonary, hepatic, renal, or haematologic disease or a severe mental health condition or other uncontrolled systemic disease; (3) alanine aminotransferase (ALT) or aspartate aminotransferase (AST) levels >1.5 times the upper normal limit, blood urea nitrogen (BUN) levels >1.2 times the upper normal limit or serum creatinine levels higher than the upper normal limit; (4) suspected allergies to the study drugs; (5) suspected or known history of alcohol/drug abuse or malignancy; (6) pregnancy or lactation; (7) participation in other clinical trials within 1 month prior to enrolment; and (8) other major concomitant diseases.

### Study Drugs, Blinding, and Randomisation

A centrally designed randomisation code was used to randomly allocate (at a 2:2:1 ratio) subjects to daily XZT, XZK, or placebo. All subjects underwent a 2-week run-in period during which they were given 2 XZT simulate capsules 3 times a day and 2 XZK simulate capsules twice a day. After that, each subject was given identically appearing capsules daily in the 12-week study period [13]: 2450-mg XZT capsules 3 times a day and 2 XZK simulate capsules twice a day in the XZT group; 2300-mg XZK capsules twice a day and 2 XZT simulate capsules 3 times a day in the XZK group; 2 XZT simulate capsules 3 times a day and 2 XZK simulate capsules twice a day in the placebo group. The study medication was labelled with sequential randomisation numbers, and each patient was assigned the lowest number available at each site at the randomisation visit. XZK and placebo provided by Dongfang Pharmaceutical Co. Ltd. (Jilin, China) (batch #20161101), XZK was obtained from Beijing WBL Peking University Biotechnology Co. Ltd. (Beijing, China) (batch #20160717/20161101).

### Concomitant Medications

Treatment with any lipid-lowering therapy (including traditional Chinese medicine and Western medicine) and/or investigational agent during the run-in period and study period was prohibited. Also excluded were medications that could affect lipid metabolism.

### Assessments

#### Endpoints

Blood samples were obtained from the cubital vein at both baseline and each monthly visit through treatment week 12 after 12 h-fasting. All samples were subsequently stored at −80 °C and analysed immediately after thawing. The concentrations of TG, TC, HDL-C, LDL-C were measured enzymatically in the laboratory of each centre using a Hitachi Automatic Biochemistry Analyser 7600 (Hitachi, Tokyo, Japan). The primary efficacy endpoints were the mean percentage changes in serum TG from baseline to week 12 (or last observation carried forward). Secondary endpoints included the success of lipid control, and the percent changes in TC, HDL-C and LDL-C from baseline to week 12. We also determined the proportions of patients whose serum lipid levels were reduced to normal at week 12 by treatment group. The assessments of safety and tolerability were based on spontaneous reports of adverse events, vital signs, and laboratory measurements.

#### Sample Size

In this study, the sample size was estimated by taking the change rate of TG as the main efficacy index. According to the results reported in the previous literature [14, 15], it is assumed that the change rate of TG before and after treatment in the positive drug control group is 22 higher than that in the placebo group, and the change rate of TG in the test group and the positive drug control group is the same, with the standard deviation of 23.5. According to the superiority cut-off value of the positive drug control group and the placebo group, the non-inferiority cut-off value of the test group and the positive drug control group is 10, α is unilateral 0.025, and β is 0.01. The sample sizes of the test group, the positive drug control group and the placebo group were 120 cases, 120 cases and 60 cases, respectively. Moreover, considering a dropout rate of approximately 20% for randomized patients, a total of 360 patients (144 in the XZT group, 144 in the XZK group, 72 in the placebo group) needed to be randomized to achieve the required number of patients for the efficacy analysis.

#### Statistical Analysis

All statistical analyses were performed with SAS software, version 9.4 (SAS Institute, Cary, North Carolina). The baseline data from the patients who underwent randomisation were analysed as the full analysis set (FAS). The primary and second efficacy endpoints were analysed in the FAS. Continuous data are presented as the mean ± standard deviation (SD), and dichotomous data are presented as numbers and percentages. The ANOVA or Kruskal-Wallis rank sum test was used to compare the measurement data, and the chi-square test or Fisher’s exact test was used to compare count data. All statistical tests were two-sided, *P* < 0.05 was considered statistically significant, and Bonferroni correction (α’ = α/n) was used in the pairwise comparison, *P* < 0.0167 was considered statistically significant.

## Results

### Patient Disposition

**Fig. 1:**
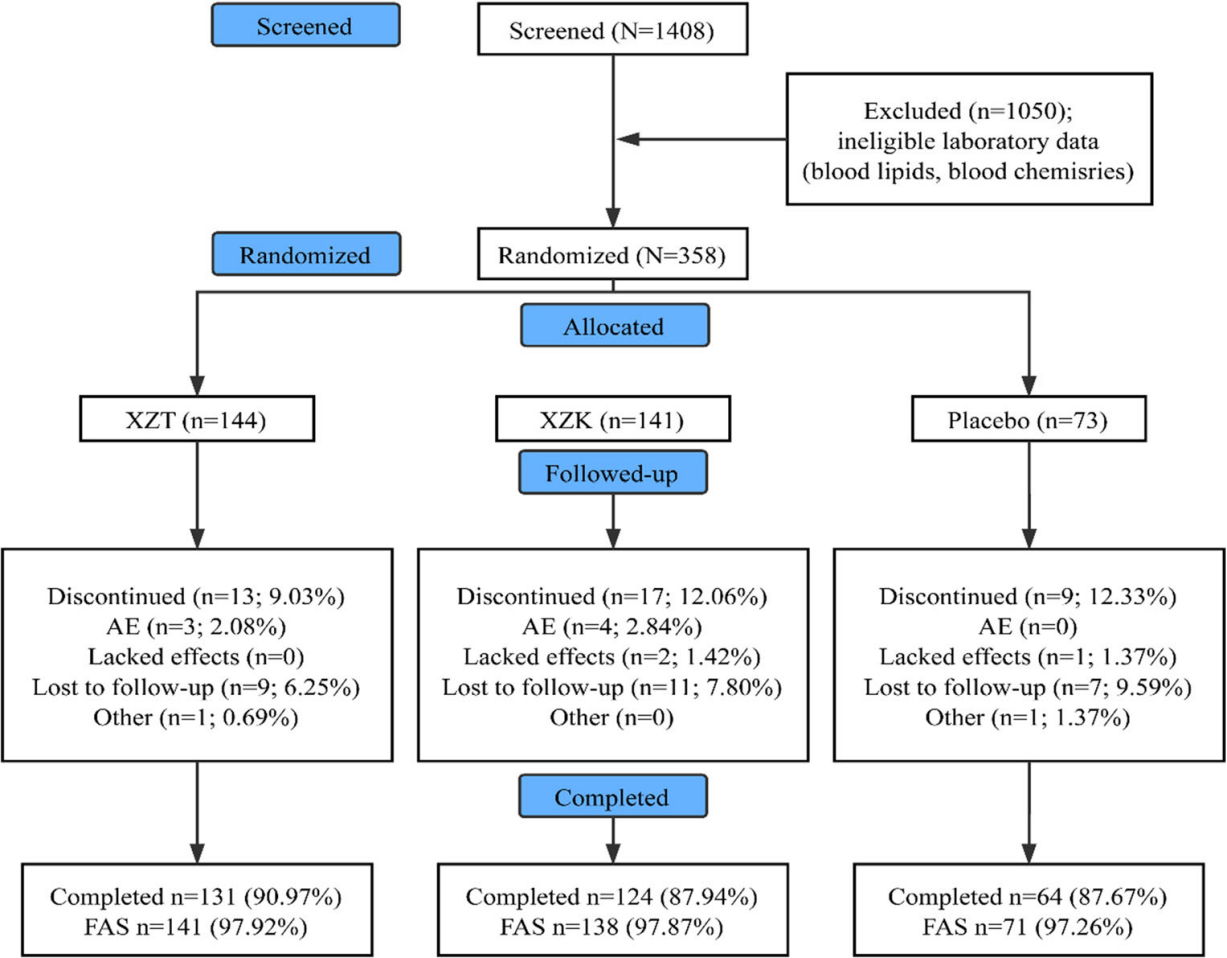
Consolidated standards of reporting trials patient disposition flow diagram. AE, adverse event; FAS, full analysis set; XZT, xuezhitong; XZK, xuezhikang

Of the 1408 patients screened, 358 had eligible blood lipids/lipoproteins and blood chemistries and were randomly allocated to the XZT (*n* = 144), XZK (*n* = 141), or placebo (*n* = 73) group (Fig. 1). A total of 39 subjects discontinued the study prematurely, including 3 subjects (2.08%) with adverse events in the XZT group and 4 subjects (2.84%) in the XZK group. Hence, 319 subjects (89.11%) completed the study, and 350 subjects (97.77%) were included in the FAS (Fig. 1).

### Baseline Characteristics

The mean (SD) ages were 51.33 (12.58), 52.54 (12.06) and 50.56 (11.72) years in the three groups, and the proportion of males in each group was close to 50%. The distributions of the demographic and clinical characteristics among the XZT group, the XZK group and the placebo group were well balanced and homogeneous (Table 2).

**Table 2:** Baseline characteristics

Characteristic	XZT (n = 141)	XZK (*n* = 138)	Placebo (n = 71)	Statistics	*P*
Demographics					
Age, years	51.33 ± 12.58	52.54 ± 12.06	50.56 ± 11.72	*F* = 0.7011	0.4968
Male	76(53.90)	75(54.35)	37(52.11)	*χ*^*2*^ = 0.0975	0.9524
Race					
Han	139(98.58)	137(99.28)	70(98.59)	Fisher	1.0000
Other	2(1.42)	1(0.72)	1(1.41)	Fisher	1.0000
Measurements					
Height, cm	166.11 ± 7.91	166.52 ± 7.42	166.77 ± 7.38	*KW-χ*^*2*^ = 0.1394	0.9327
Weight, kg	70.10 ± 11.19	70.72 ± 10.95	73.03 ± 12.03	*KW-χ*^*2*^ = 2.4450	0.2945
BMI, kg/m^2^	25.30 ± 2.72	25.40 ± 2.75	26.16 ± 3.10	*F* = 2.4439	0.0883
Systolic BP, mm Hg	127.13 ± 8.94	127.28 ± 9.83	126.62 ± 8.79	*KW-χ*^*2*^ = 0.7230	0.6966
Diastolic BP, mm Hg	76.91 ± 6.81	77.43 ± 8.11	77.65 ± 7.89	*KW-χ*^*2*^ = 0.2274	0.8925
Heart rate, beats/min	71.99 ± 7.34	70.62 ± 7.56	70.28 ± 8.64	*KW-χ*^*2*^ = 2.4704	0.2908
History of lipid-lowering drug treatment	19(13.84)	25(18.12)	18(25.35)	*χ*^*2*^ = 4.5951	0.1011
Medical history					
Hypertension	57(40.43)	48(34.78)	37(52.11)	*χ*^*2*^ = 5.8416	0.0552
Diabetes mellitus	31(21.99)	25(18.12)	16(22.54)	*χ*^*2*^ = 0.8494	0.6603
Stable CAD	17(12.06)	7(5.07)	7(9.86)	*χ*^*2*^ = 4.3250	0.1188
Laboratory measurements					
Fasting blood glucose, mmol/L	6.11 ± 1.41	6.23 ± 1.85	5.94 ± 1.18	*KW-χ*^*2*^ = 0.5914	0.7440
TG, mmol/L	3.40 ± 0.93	3.28 ± 0.91	3.41 ± 0.87	*KW-χ*^*2*^ = 2.1298	0.3448
TC, mmol/L	6.21 ± 0.59	6.17 ± 0.67	5.98 ± 0.59	*KW-χ*^*2*^ = 2.0465	0.3594
LDL-C, mmol/L	3.82 ± 0.47	3.82 ± 0.52	3.82 ± 0.56	*KW-χ*^*2*^ = 0.1107	0.9461
HDL-C, mmol/L	0.88 ± 0.14	0.88 ± 0.17	0.87 ± 0.13	*F* = 0.0465	0.9546

Values are mean ± SD, %, n (%). BMI, body mass index; XZT, xuezhitong; XZK, xuezhikang

CAD, coronary artery disease; TG, triglyceride; TC, total cholesterol; LDL-C, low-density lipoprotein cholesterol; HDL-C, high-density lipoprotein cholesterol

### Efficacy

**Fig. 2:**
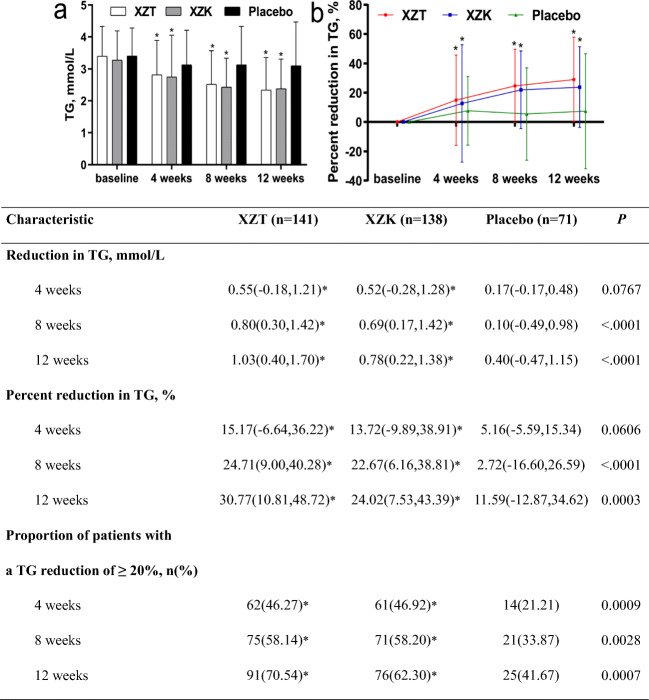
**TG change from baseline to each treatment timepoint.** Values are the median (Q1, Q3) or the % of patients. Reduction in TG = baseline level – 4-, 8-, and 12-week levels. Percent reduction in TG = (baseline level - 4, 8, 12 weeks level) / baseline level × 100%. *P < 0.0167 for active treatment compared with placebo

Daily treatment with XZT and XZK significantly improved both the reduction in TG and the percent reduction in TG from baseline to treatment weeks 4, 8 and 12 (each *P* < 0.05 vs baseline and vs placebo; Fig. 2). However, there was no significant difference between the XZT and XZK treatments at each visit point from baseline to treatment weeks 4, 8 and 12. After 12 weeks of treatment, both groups showed a significant increase in the proportion of patients with a TG reduction of ≥20% from baseline, but treatment with XZT and XZK led to a significantly greater reduction than did the placebo (each *P* < 0.05 vs placebo), and the XZT group had better results than the XZK group (70.54% vs 62.30%; *P* = 0.166).

Significant changes were also observed across many secondary efficacy variables after 12 weeks of treatment. XZT and XZK enabled approximately 20% (17.97%, 19.67%) of the subjects to successfully control their lipids compared with baseline (each *P* < 0.0167 vs placebo; *P* = 0.730 for XZT and XZK comparisons) (Table 3). In the XZT and XZK groups, the changes in the reduction and the percent reduction in TC were significantly greater than those in the placebo group (*P* < 0.0167), but there was no significant difference between the two groups. The XZK group performed better than the XZT group in terms of the proportion of patients with a TC reduction ≥10% (85.29% vs 58.14%; *P* < 0.1675). Both the XZT and XZK groups showed a greater reduction in LDL-C, greater percent reduction in LDL-C, and a higher proportion of patients with an LDL-C reduction ≥10% compared with the placebo group (each *P* < 0.0167). The HDL-C level increased significantly from baseline to week 12 with XZT (*P* < 0.0167 vs placebo). A total of 65.22% of the patients in the XZT group had an increase in HDL-C levels of at least 4 mg/dl, compared with 38.30% of patients in the XZK group (*P* < 0.0167) and 25.00% of patients in the placebo group (*P* < 0.0167).

**Table 3:** Efficacy of treatments for secondary endpoints

Characteristic	XZT (*n* = 141)	XZK (*n* = 138)	Placebo (*n* = 71)	Statistics	*P*
Success rate of lipid control, %	17.97*	19.67*	5.00	*χ*^*2*^ = 6.9459	0.0310
Reduction in TC, mmol/L	0.88 ± 1.02*	1.20 ± 0.86*	0.24 ± 0.48	*KW-χ*^*2*^ = 12.5803	0.0019
Percent reduction in TC, %	14.18 ± 16.13*	19.05 ± 13.15*	3.89 ± 8.14	*KW-χ*^*2*^ = 12.6346	0.0018
Proportion of patients with a TC reduction ≥ 10%, %	58.14†	85.29*	33.33	*χ*^*2*^ = 13.4758	0.0012
Reduction in LDL-C, mmol/L	0.72 ± 0.62*	0.88 ± 0.78*	0.12 ± 0.47	*F* = 8.4454	0.0004
Percent reduction in LDL-C, %	17.98 ± 15.70*	22.33 ± 18.03*	2.95 ± 12.78	*F* = 9.0975	0.0002
Proportion of patients with an LDL-C reduction ≥ 10%, %	80.00*	80.95*	33.33	Fisher	0.0005
Increase in HDL-C, mmol/L	0.17 ± 0.18*	0.12 ± 0.27	0.01 ± 0.11	*KW-χ*^*2*^ = 14.2014	0.0008
Percent increase in HDL-C, %	21.47 ± 24.00*	17.95 ± 35.69	2.16 ± 12.63	*KW-χ*^*2*^ = 12.7730	0.0017
Proportion of patients with an HDL-C increase ≥ 4 mg/dl, %	65.22*†	38.30	25.00	*χ*^*2*^ = 12.9423	0.0016

Values are expressed as the mean ± SD or the % of patients. Reduction in TC, LDL-C = baseline level - 12 weeks level. Percent reduction in TC, LDL-C = (baseline level - 12 weeks level) / baseline level × 100%. Reduction in HDL-C = 12 weeks level - baseline level. Percent reduction in HDL-C = (12 weeks level - baseline level) / baseline level × 100%. *P < 0.0167 for active treatment compared with placebo; †*P* < 0.0167 for XZT compared with XZK

### Tolerability and Safety

A total of 350 patients in 3 groups were included in the safety set analyses (Table 4). Treatment with XZT was well tolerated and had tolerability profiles similar to those of the XZK and placebo groups. Similar proportions of patients in the XZK and placebo groups reported adverse events. Most adverse events in clinical drug research are gastrointestinal (GI), and these were considered to be mild or moderate in this study. One subject in the XZT group experienced myalgia but had no evidence of myopathy (specified in the protocol as muscle pain accompanied by an increase in CK to ≥10 × ULN). Some adverse events may have been related to the study drugs: (1) abdominal pain, abnormal AST, abnormal urine protein, haemoglobin reduction and cutaneous effects (rash) were observed in 1 subject each in the XZT group; (2) abnormal liver transaminases along with anaemia or hyperbilirubinemia were observed in 2 subjects in the XZK group, and increased CK increased and headache were reported by two others; and (3) abdominal pain was reported by 1 subject in the placebo group. Most of these subjects withdrew due to adverse events. The analysis of drug-induced adverse events and withdrawal revealed no differences between the study groups.

**Table 4:** Adverse events

	XZT(*n* = 141)	XZK(*n* = 138)	Placebo (*n* = 71)	*P*
	n (cases), %	n (cases), %	n (cases), %	
AEs	23(28), 16.31	33(41), 23.91	15(19), 21.13	0.2821
Gastrointestinal				
Dyspepsia	1, 0.71	0	1, 1.41	
Diarrhoea	0	2, 1.45	0	
Abdominal discomfort	0	2, 1.45	0	
Investigations/laboratory abnormalities				
Increased AST	0	1, 0.72	1, 1.41	
Increased ALT	1, 0.71	1, 0.72	1, 1.41	
Increased CK	0	1, 0.72	1, 1.41	
Increased leukocyte count	1, 0.71	1, 0.72	1, 1.41	
Increased Cr	0	1, 0.72	1, 1.41	
Increased Bun	0	0	0	
Infections				
Upper-respiratory tract infection	1, 0.71	3, 2.17	4, 5.63	
Nervous system disorder				
Headache	0	1, 0.72	0	
Musculoskeletal and connective-tissue disorders				
Muscle spasm	0	0	0	
Myalgia	1, 0.71	0	0	
AEs related to the study drugs	5(5), 3.55	4(4), 2.90	0	0.8461
SAEs	0	0	1(1), 1.41	0.2029
Withdrawal due to adverse events	3(3), 2.13	4(4), 2.90	0	0.4787

The analysis included all patients who received at least 1 dose of the study drugs. Some patients reported more than 1 event. AEs, adverse events; SAEs, serious adverse events; ALT, alanine aminotransferase; AST, aspartate aminotransferase; CK, creatine kinase; Cr, creatinine; Bun, blood urea nitrogen

There were no clinically meaningful differences between the treatment groups in the laboratory test, vital signs, or physical examination results. No subject exhibited ≥2-fold elevations in CK or liver transaminases. One subject experienced serious (non-drug-related) adverse events: a patient in the placebo group had symptoms of chest distress, panic, dizziness and fatigue, with no change in her study medication.

## Discussion

According to an epidemiological survey of China in 2010, among 97,409 residents over 18 years old, the prevalence rates of HTG, low HDL-C and high LDL-C were 11.3%, 44.8%, and 2.1%, respectively, [16]. The DYSIS study, which covers 6 regions, 27 provinces and 122 hospitals in China, included 25,317 patients who had taken statins for at least 3 months. The results showed that there were still as many as 47.6% of patients with HTG and/or low HDL-C; in the very high-risk group (patients were considered to be those with CHD or ischaemic stroke plus diabetes, or acute coronary syndrome), the proportion was as high as 74.2% [17]. Persistent hypertriglyceridaemia indicates a significant residual cardiovascular risk, even in patients with well-controlled LDL cholesterol levels achieved by high-intensity statin regimens [18]. Currently, some components are available that can decrease TG, namely, fibrates, Omega-3 fatty acids, and nicotinic acid [19]. However, high-purity Omega-3 fatty acids products are not clinically available, and nicotinic acid drugs may raise blood sugar in patients [20]. It is urgent to find and research new TG-lowering drugs.

XZT is synthesized from Allium macrostemon extract. It has proven that Allium macrostemon contains methyl allyl trisulfide, macrostemonoside A and other sulphur compounds, which have the effect of lipid lowering and anti-atherosclerosis [8]. The pharmacological action of XZT on the cardiovascular system is to reduce the levels of TC, TG and LDL-C in the blood, increase the level of HDL-C, inhibit the aggregation of platelets and thrombosis, protect against myocardial injury caused by hypoxia ischaemia and ischaemia-reperfusion, and prevent and control atherosclerosis at the same time [21]. Daily treatment with XZT and XZK resulted in statistically and clinically significant improvement from baseline to week 12 in terms of the reduction of TG and the percent reduction of TG. XZT and XZK enabled approximately 70% of the subjects to achieve ≥20% reductions in TG level from baseline. XZT and XZK also significantly improved the success rate of lipid control and improved the levels of other atherogenic lipids, such as the reduction/increase, percent reduction/increase and effective rate of TC, LDL-C and HDL-C. Our efficacy data are largely consistent with the results from previous studies [9, 22]. Therefore, these agents may provide clinical benefits for patients with CV residual risk/CV events based on the treatment of statins, or they can be used as alternative drugs for patients with intolerance of statins.

In the present study, XZT and XZK proved to be effective in the treatment of dyslipidemia, and the mechanisms are still being explored. Zhao et al. investigated the effects of XZK on the TG level compared with simvastatin in the setting of an equivalent LDL-C lowering power, and found that XZK has stronger hypotriglyceridemic performance than simvastatin, which is attributed to more apolipoprotein A5 (apoA5) up-regulation by XZK through peroxisome proliferator-activated receptor α (PPARα) signalling pathway [23]. Our present study demonstrated that XZT and XZK have similar performance of reducing TG and LDL-C levels, but XZT has stronger ability to increase HDL-C level. HDL-C, as the acceptor of cholesterol, can transport cholesterol from surrounding tissues (including macrophages and atherosclerotic plaques) to the liver for recycling or excretion in the form of cholic acid. This process is called reverse cholesterol transport (RCT), through which can reduce the cholesterol level in plasma and blood vessel wall and anti-atherosclerosis [24]. Meng et al. detected the mechanism of XZT in the treatment of dyslipidemia involving RCT. The study indicated that XZT improved blood lipid dysfunction by induced RCT activation and increased the HDL levels of high fat diet-fed ApoE^−/−^mice [25].

Similar frequencies of adverse events (chiefly GI effects) were observed in the 3 groups. Although 1 subject receiving XZT experienced muscle symptoms, none had myopathy. In previous trials, XZT proved to be safe and well tolerated among patients with pre-existing abnormal liver function tests or statin-associated myalgia. Allium macrostemon is a dual-purpose plant used for medicine and food, so the XZT extracted and synthesized from it has good tolerability and safety.

### Strengths, Limitations and Future Work

Our study had strengths and limitations. To the best of our knowledge, this is the first multicentre, randomized, double-blind, double simulation, positive drug and placebo parallel control study to evaluate the efficacy and safety of XZT using a large sample size. The 17 sites participated in our study are located in various regions of China, and the results are representative. Moreover, all patients have undergone a 2-week therapeutic lifestyle change period before inclusion, so that the enrolled patients are most in need of drug treatment, making the results of the study more reliable. In addition, XZT is extracted from a medicine food homology plant, which not only has a good efficacy of dyslipidemia, but also has a good tolerability and safety, it provides a new direction for the development of new lipid-modulating drugs.

However, there were also several limitations in our study. Firstly, this study only evaluated the lipid-modulating efficacy and safety of XZT treatment for 12 weeks, while the efficacy of the drug, especially the safety, needs to be tested for a longer time. Secondly, this study only included patients with moderate TG levels (2.3 mmol/L~5.6 mmol/L), but not patients with severe TG level (>5.6 mmol/L), which may make the results biased. Thirdly, HTG is closely related to residual cardiovascular risk, and the present study only evaluated the effect of XZT on triglyceride-lowering performance, but the effect among patients who have achieved statins therapy on residual cardiovascular risk is not available here. Lastly, patients with type 2 diabetes are often associated with HTG, but there is no further subgroup study on diabetes in this study. Moreover, only one dose of each drug was tested and dose response curves have not been performed. 

Future study should enlarge the sample size, include with sever HTG patients with well-controlled LDL-C levels by statins, and each patient treated for an extended period (at least 1.5~2 years), and observe the clearer primary endpoint (CV death, MI, stroke, hospitalization for unstable angina requiring unplanned revascularization) to evaluate the efficacy and safety of XZT on residual cardiovascular risk [26]. Most of all, more studies are needed to clarify the possible mechanism involved in the lipid-modulating effect of XZT.

## Conclusions

Treatment with XZT daily for 12 weeks significantly reduced levels of TG and other atherogenic lipids and was safe and well tolerated. Further prospective randomized controlled trials are warranted to evaluate the efficacy, safety, and tolerability of XZT in larger, more clinically heterogeneous patient populations followed for longer intervals.
